# Crystal structure of 1,2,3,4-tetra­hydro­isoquinolin-2-ium (2*S*,3*S*)-3-carb­oxy-2,3-di­hydroxy­propano­ate monohydrate

**DOI:** 10.1107/S2056989024005711

**Published:** 2024-06-21

**Authors:** Rüdiger W. Seidel, Tsonko M. Kolev

**Affiliations:** a Institut für Pharmazie, Martin-Luther-Universität Halle-Wittenberg, Wolfgang-Langenbeck-Str. 4, 06120 Halle (Saale), Germany; b Institute of Molecular Biology "Roumen Tsanev", Bulgarian Academy of Sciences, Acad. G. Bonchev-Str. Bl. 21, Sofia 1113, Bulgaria; University of Neuchâtel, Switzerland

**Keywords:** iso­quinoline, tartaric acid, salt formation, proton-transfer compound, hydrogen bonding, crystal structure

## Abstract

The crystal structure of 1,2,3,4-tetra­hydro­isoquinolin-2-ium (2*S*,3*S*)-3-carb­oxy-2,3-di­hydroxy­propano­ate monohydrate, C_9_H_12_N^+^·C_4_H_5_O_6_^−^·H_2_O, at 115 K shows ortho­rhom­bic symmetry (space group *P*2_1_2_1_2_1_). The hydrogen tartrate anions and solvent water mol­ecules form an intricate diperiodic O—H⋯O hydrogen-bond network parallel to (001). The tetra­hydro­isoquinolinium cations are tethered to the anionic hydrogen-bonded layers through N—H⋯O hydrogen bonds. The crystal packing in the third direction is achieved through van der Waals contacts between the hydro­carbon tails of the tetra­hydro­isoquinolinium cations, resulting in hydro­phobic and hydro­philic regions in the crystal structure.

## Abstract 2

The crystal structure of 1,2,3,4-tetra­hydro­isoquinolin-2-ium (2*S*,3*S*)-3-carb­oxy-2,3- di­hydroxy­propano­ate monohydrate (ortho­rhom­bic crystal system, space group *P*2_1_2_1_2_1_, *Z* = 4) features an intricate two-dimensional hydrogen-bond network.

## 1. Chemical context

1,2,3,4-Tetra­hydro­iso­quinoline is a secondary amine derived from iso­quinoline. Tetra­hydro­iso­quinoline alkaloids represent a large and structurally diverse group of natural products with a wide range of biological activity (Kim *et al.*, 2023 ▸). The tetra­hydro­iso­quinoline scaffold is also encountered in a number of approved drugs, for example in the angiotensin-converting-enzyme inhibitor quinapril and in the anti­muscarinic solifenacin. Thus far, few salts of 1,2,3,4-tetra­hydro­iso­quinoline have been structurally characterized (see Section 4). Herein, we describe the crystal and mol­ecular structure of 1,2,3,4-tetra­hydro­isoquinolinium hydrogen tartrate monohydrate [systematic name: 1,2,3,4-tetra­hydro­isoquinolin-2-ium (2*S*,3*S*)-3-carb­oxy-2,3-di­hydroxy­propano­ate hydrate]. Hydrogen tartrate is a well-known anion in pharmaceutics (Bharate, 2021 ▸). The p*K*_a_ of the conjugate acid of tetra­hydro­iso­quinoline is 9.3 (at 310 K; Bojarski *et al.*, 1995 ▸), and the p*K*_a1_ of tartaric acid is 2.9 (at 298 K; Dawson, 1959 ▸). According to the p*K*_a_ rule (Cruz-Cabeza, 2012 ▸), we can estimate Δp*K*_a_ = p*K*_a_[protonated base] − p*K*_a_[acid] = 9.3 – 2.9 = 6.4. Hence, proton transfer is expected when tetra­hydro­iso­quinoline is reacted with tartaric acid.
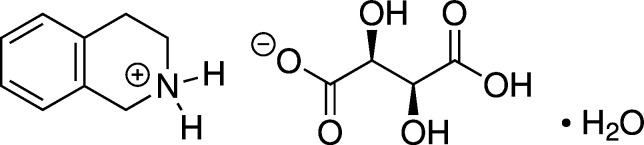


## 2. Structural commentary

**Figure 1:**
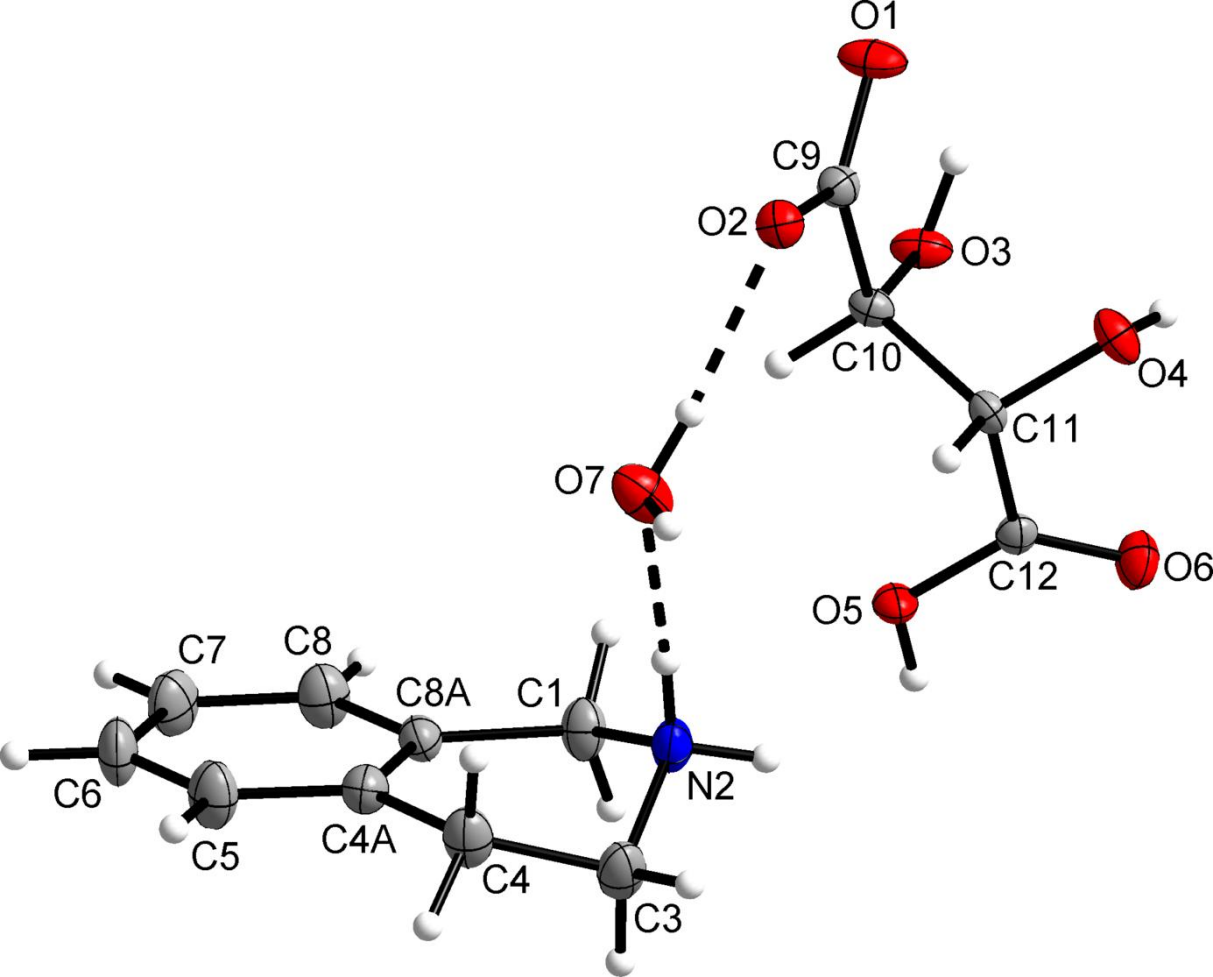
The asymmetric unit of the title compound with displacement ellipsoids at the 50% probability level. Hydrogen atoms are presented by small spheres of arbitrary radius. Dashed lines illustrate hydrogen bonds.

Fig. 1 ▸ shows a displacement ellipsoid plot of the mol­ecular components of the salt in the solid state. The asymmetric unit comprises a 1,2,3,4-tetra­hydro­isoquinolin-2-ium cation, a (2*S*,3*S*)-hydrogen tartrate anion and a water mol­ecule of crystallization. The axially chiral conformation of the tetra­hydro­isoquinolinium cation is left-handed, as revealed by the C4—C3—N2—C1 torsion angle of −65.8 (3)°. The carbon skeleton of the hydrogen tartrate anion adopts an anti­periplanar (*anti*) conformation [C9—C10—C11—C12 = 178.67 (15)°], which is known to be the predominant one in tartaric acid derivatives (Gawronski *et al.*, 2005 ▸). The carb­oxy group of the anion exhibits the *syn* conformation.

## 3. Supra­molecular features

**Figure 2:**
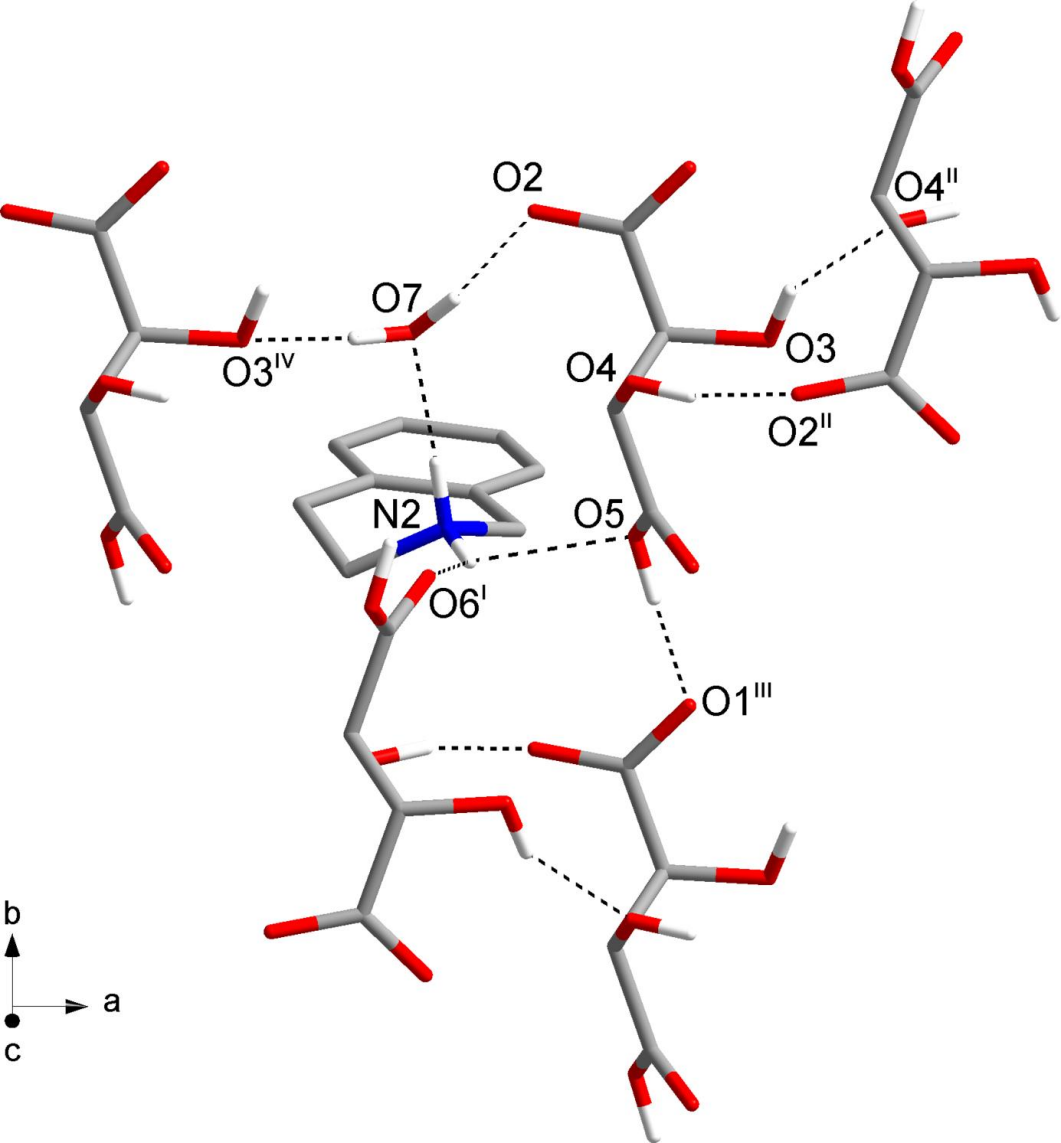
Section of the crystal structure, viewed along the [011] direction, showing the unique hydrogen bonds (dashed lines). Symmetry codes: (i) *x* − 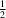, −*y* + 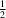, −*z* + 1; (ii) *x* + 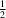, −*y* + 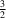, −*z* + 1; (iii) *x*, *y* − 1, *z*; (iv) *x* − 1, *y*, *z*.

**Figure 3:**
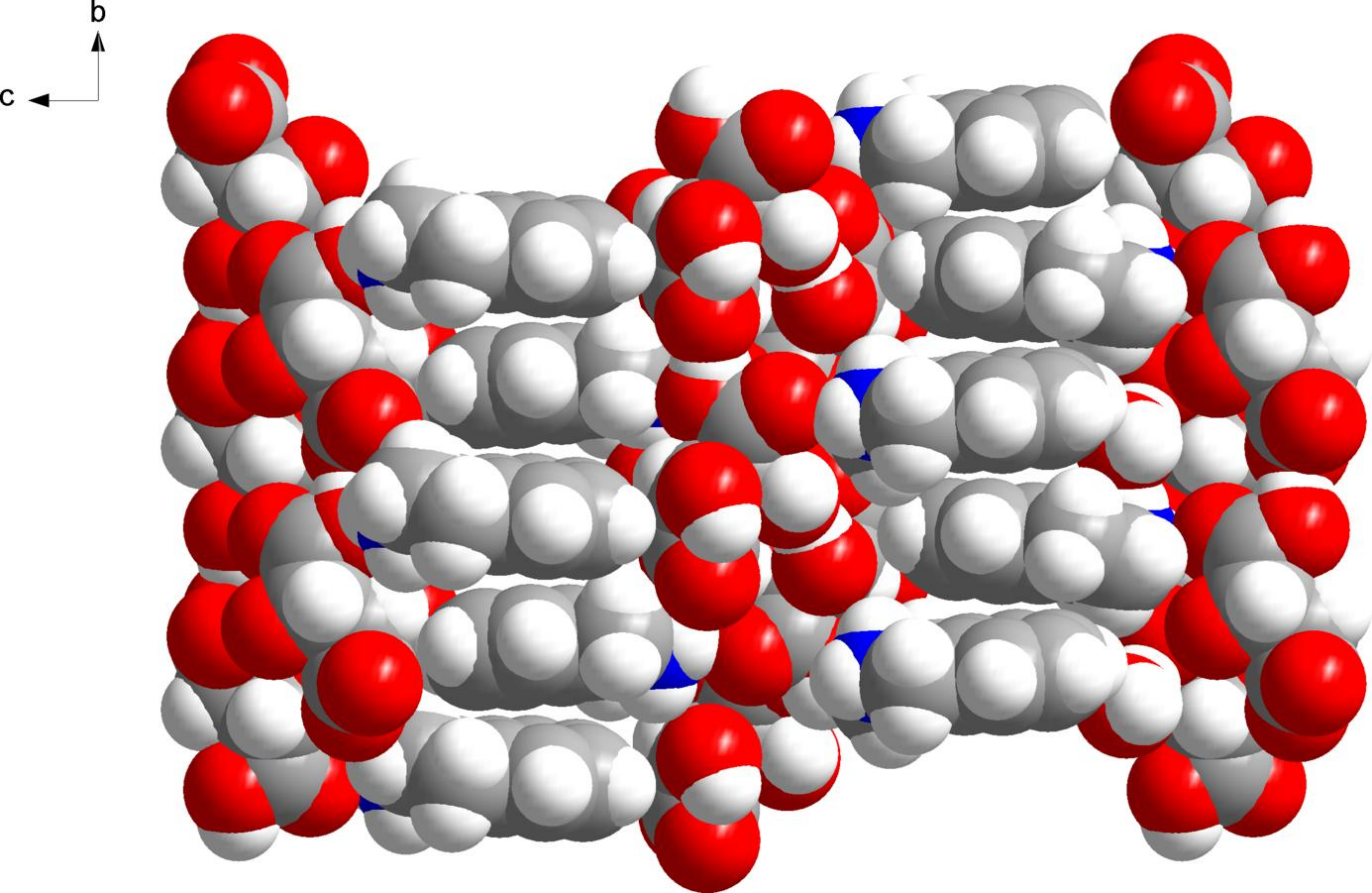
Space-filling representation of the crystal structure, viewed along the *a*-axis direction. Colour scheme: C, grey; H, white; N, blue; O, red.

The solid state supra­molecular structure features an intricate network of N—H⋯O and O—H⋯O hydrogen bonds (Fig. 2 ▸). Table 1 ▸ lists the corresponding geometric parameters, which are within expected ranges (Thakuria *et al.*, 2017 ▸). The hydrogen tartrate anions form hydrogen-bonded chains by translational symmetry in the *b-*axis direction through hydrogen bonding between the carb­oxy group and the carboxyl­ate group of an adjacent mol­ecule (O5—H5*A*⋯O1^iii^). In the *a-*axis direction, the hydrogen tartrate ions are connected along a 2_1_ screw axis *via* two hydrogen bonds with the two hy­droxy groups as donors and a hy­droxy group (O3—H3⋯O4^ii^) and the carboxyl­ate group (O4—H4⋯O2^ii^) of a neighbouring mol­ecule as acceptors. These O—H⋯O hydrogen-bonding inter­actions that extend in the *a-* and *b-*axis directions result in diperiodic hydrogen-bonded sheets parallel to (001). The protonated amino group of the tetra­hydro­isoquinolinium cation forms a bifurcated hydrogen bond to the carb­oxy groups of two adjacent hydrogen tartrate anions (N2—H2*B*⋯O5 and N2—H2*B*⋯O6^i^) and another hydrogen bond to the solvent water mol­ecule (N2—H2*A*⋯O7). The water mol­ecule in turn acts as a hydrogen-bond donor towards the carboxyl­ate group (O7—H*A*⋯O2) and a hy­droxy group (O7—H*B*⋯O3^iv^) of two hydrogen tartrate anions. The hydro­carbon parts of the tetra­hydro­isoquinolinium cations are oriented approximately perpendicular to the diperiodic hydrogen-bonded sheets formed by the hydrogen tartrate anions. The crystal packing in the third dimension is achieved by stacking in the *c*-axis direction with inter­locking of the hydro­carbon tails through van der Waals packing (Fig. 3 ▸). This affords hydro­phobic and hydro­philic regions in the crystal structure.

## 4. Database survey

A survey of the Cambridge Structural Database (CSD, version 5.43, update of September 2022; Groom *et al.*, 2016 ▸) revealed that crystal structures of salts of tetra­hydro­isoquinolinium are scarce. Thus far, the crystal structures of a solvent-free hydro­chloride (CSD refcode: GESVOR; Zia-ur-Rehman *et al.*, 2012 ▸), hydrogen squarate (TIGKIE; Kolev *et al.*, 2007 ▸), hexa­chlorido­stannate(IV) (AYAHAM; Dhanalakshmi *et al.*, 2021 ▸) and hexa­bromido­stannate(IV) (AYAHEQ; Dhanalakshmi *et al.*, 2021 ▸) as well as a violurate monohydrate (FUFPOM; Kolev *et al.*, 2009 ▸) have been reported. The solid-state structure of free-base tetra­hydro­isoquinolinium, which is liquid at ambient conditions, is hitherto unknown, as far as we are able to ascertain. In contrast, hundreds of crystal structures containing hydrogen tartrate anions can be found in the CSD. In the vast majority of these crystal structures, the carbon skeleton of the hydrogen tartrate anion exhibits the *anti* conformation. Exceptions are the crystal structure of quininium (*S*,*S*)-hydrogen tartrate hemihydrate (PUVTUV; Ryttersgaard & Larsen, 1998 ▸), lithium *meso*-hydrogen tartrate monohydrate (COFGAF10; Stouten *et al.*, 1988 ▸), potassium *meso*-hydrogen tartrate monohydrate (KHMTAR01; Currie *et al.*, 1975 ▸) and 1-(4′-cyano-4′-cyclo­hexyl-4′-phenyl­but­yl)piperidinium (*S*,*S*)-hydrogen tartrate (EZOWUL; Jones, 2004 ▸) in which the hydrogen tartrate anions are found in the *gauche* conformation.

## 5. Synthesis and crystallization

Starting materials were obtained from commercial sources and used as received. A mixture of 1,2,3,4-tetra­hydro­iso­quinoline (266 mg, 2 mmol) and excess (2*S*,3*S*)-tartaric acid (1.50 g, 10 mmol) in 60 mL of deionized water was stirred for four h at room temperature. Subsequently, the salt was isolated by filtration. Colourless crystals suitable for single-crystal X-ray diffraction were obtained from a water/methanol (3:1) solution of the salt, after the solvents were allowed to evaporate slowly at ambient conditions.

## 6. Refinement

Crystal data, data collection and structure refinement details are summarized in Table 2 ▸. Carbon-bound hydrogen atoms were placed in geometrically calculated positions and refined using the appropriate riding model with C—H_aromatic_ = 0.95 Å, C—H_methyl­ene_ = 0.99 Å, C—H_methine_ = 1.00 Å and *U*_iso_(H) = 1.2 *U*_eq_(C). Nitro­gen- and oxygen-bound hydrogen atoms were located in difference-Fourier maps and subsequently refined semi-freely with the N—H and the O—H distances restrained to target values of 0.88 (2) Å and 0.84 (2) Å, respectively.

## Supplementary Material

Crystal structure: contains datablock(s) I, global. DOI: 10.1107/S2056989024005711/tx2087sup1.cif

Structure factors: contains datablock(s) I. DOI: 10.1107/S2056989024005711/tx2087Isup2.hkl

Supporting information file. DOI: 10.1107/S2056989024005711/tx2087Isup3.cdx

Supporting information file. DOI: 10.1107/S2056989024005711/tx2087Isup4.cml

CCDC reference: 2362561

Additional supporting information:  crystallographic information; 3D view; checkCIF report

## Figures and Tables

**Table 1:** Hydrogen-bond geometry (Å, °)

*D*—H⋯*A*	*D*—H	H⋯*A*	*D*⋯*A*	*D*—H⋯*A*
N2—H2*A*⋯O7	0.91 (2)	1.85 (2)	2.762 (3)	176 (2)
N2—H2*B*⋯O6^i^	0.88 (2)	2.09 (2)	2.787 (2)	135 (2)
N2—H2*B*⋯O5	0.88 (2)	2.41 (2)	2.981 (2)	123 (2)
O3—H3⋯O4^ii^	0.87 (2)	1.98 (2)	2.766 (2)	151 (3)
O4—H4⋯O2^ii^	0.82 (2)	1.99 (2)	2.730 (2)	150 (3)
O5—H5*A*⋯O1^iii^	0.89 (2)	1.60 (2)	2.480 (2)	173 (3)
O7—H7*A*⋯O2	0.87 (2)	1.91 (2)	2.782 (2)	173 (3)
O7—H7*B*⋯O3^iv^	0.87 (2)	1.92 (2)	2.772 (2)	169 (3)

Symmetry codes: (i) 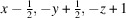; (ii) 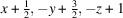; (iii) 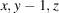; (iv) 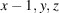.

**Table 2:** Experimental details

Crystal data	
Chemical formula	C_9_H_12_N^+^·C_4_H_5_O_6_^−^·H_2_O
*M*_r_	301.29
Crystal system, space group	Orthorhombic, *P*2_1_2_1_2_1_
Temperature (K)	115
*a*, *b*, *c* (Å)	7.0695 (3), 7.4842 (3), 26.9573 (10)
*V* (Å^3^)	1426.30 (10)
*Z*	4
Radiation type	Mo *K*α
μ (mm^−1^)	0.12
Crystal size (mm)	0.49 × 0.21 × 0.20

Data collection	
Diffractometer	Oxford Diffraction Xcalibur2
Absorption correction	Multi-scan (ABSPACK in *CrysAlis PRO*; Rigaku OD, 2023 ▸)
*T*_min_, *T*_max_	0.967, 1.000
No. of measured, independent and observed [*I* > 2σ(*I*)] reflections	16488, 3307, 2900
*R*_int_	0.038
(sin θ/λ)_max_ (Å^−1^)	0.671

Refinement	
*R*[*F*^2^ > 2σ(*F*^2^)], *wR*(*F*^2^), *S*	0.037, 0.078, 1.04
No. of reflections	3307
No. of parameters	218
No. of restraints	7
H-atom treatment	H atoms treated by a mixture of independent and constrained refinement
Δρ_max_, Δρ_min_ (e Å^−3^)	0.25, −0.19
Absolute structure	The absolute structure was inferred from the known absolute configuration of the starting material.

Computer programs: *CrysAlis* system (Oxford Diffraction, 2009 ▸), *CrysAlis PRO* (Rigaku OD, 2023 ▸), *SHELXS97* (Sheldrick, 2008 ▸), *SHELXL2019/3* (Sheldrick, 2015 ▸), *DIAMOND* (Brandenburg, 2018 ▸) and *publCIF* (Westrip, 2010 ▸).

## supplementary crystallographic information

### 1,2,3,4-Tetrahydroisoquinolin-2-ium (2S,3S)-3-carboxy-2,3-dihydroxypropanoate monohydrate Crystal data

**Table:**

C_9_H_12_N^+^·C_4_H_5_O_6_^−^·H_2_O	*D*_x_ = 1.403 Mg m^−^^3^
*M**_r_* = 301.29	Mo *K*α radiation, λ = 0.71073 Å
Orthorhombic, *P*2_1_2_1_2_1_	Cell parameters from 5780 reflections
*a* = 7.0695 (3) Å	θ = 3.0–27.7°
*b* = 7.4842 (3) Å	µ = 0.12 mm^−^^1^
*c* = 26.9573 (10) Å	*T* = 115 K
*V* = 1426.30 (10) Å^3^	Prism, colourless
*Z* = 4	0.49 × 0.21 × 0.20 mm
*F*(000) = 640	

### 1,2,3,4-Tetrahydroisoquinolin-2-ium (2S,3S)-3-carboxy-2,3-dihydroxypropanoate monohydrate Data collection

**Table:**

Oxford Diffraction Xcalibur2 diffractometer	3307 independent reflections
Radiation source: fine-focus sealed X-ray tube, Enhance (Mo) X-ray Source	2900 reflections with *I* > 2σ(*I*)
Graphite monochromator	*R*_int_ = 0.038
Detector resolution: 8.4171 pixels mm^-1^	θ_max_ = 28.5°, θ_min_ = 2.8°
ω scans	*h* = −9→9
Absorption correction: multi-scan (ABSPACK in CrysAlisPro; Rigaku OD, 2023)	*k* = −9→9
*T*_min_ = 0.967, *T*_max_ = 1.000	*l* = −35→35
16488 measured reflections	

### 1,2,3,4-Tetrahydroisoquinolin-2-ium (2S,3S)-3-carboxy-2,3-dihydroxypropanoate monohydrate Refinement

**Table:**

Refinement on *F*^2^	Secondary atom site location: difference Fourier map
Least-squares matrix: full	Hydrogen site location: mixed
*R*[*F*^2^ > 2σ(*F*^2^)] = 0.037	H atoms treated by a mixture of independent and constrained refinement
*wR*(*F*^2^) = 0.078	*w* = 1/[σ^2^(*F*_o_^2^) + (0.0326*P*)^2^ + 0.2898*P*] where *P* = (*F*_o_^2^ + 2*F*_c_^2^)/3
*S* = 1.04	(Δ/σ)_max_ < 0.001
3307 reflections	Δρ_max_ = 0.25 e Å^−^^3^
218 parameters	Δρ_min_ = −0.19 e Å^−^^3^
7 restraints	Absolute structure: The absolute structure was inferred from the known absolute configuration of the starting material.
Primary atom site location: structure-invariant direct methods	

### 1,2,3,4-Tetrahydroisoquinolin-2-ium (2S,3S)-3-carboxy-2,3-dihydroxypropanoate monohydrate Special details

**Table:**

Geometry. All esds (except the esd in the dihedral angle between two l.s. planes) are estimated using the full covariance matrix. The cell esds are taken into account individually in the estimation of esds in distances, angles and torsion angles; correlations between esds in cell parameters are only used when they are defined by crystal symmetry. An approximate (isotropic) treatment of cell esds is used for estimating esds involving l.s. planes.

### 1,2,3,4-Tetrahydroisoquinolin-2-ium (2S,3S)-3-carboxy-2,3-dihydroxypropanoate monohydrate Fractional atomic coordinates and isotropic or equivalent isotropic displacement parameters (Å^2^)

**Table:**

	*x*	*y*	*z*	*U*_iso_*/*U*_eq_	
C1	0.4189 (3)	0.1694 (3)	0.34142 (7)	0.0239 (5)	
H1A	0.464782	0.045500	0.345983	0.029*	
H1B	0.528981	0.250520	0.344259	0.029*	
C3	0.0946 (3)	0.1243 (4)	0.37283 (8)	0.0285 (5)	
H3A	0.011223	0.144808	0.401784	0.034*	
H3B	0.112372	−0.006161	0.368861	0.034*	
C4	0.0045 (3)	0.2016 (4)	0.32647 (8)	0.0270 (6)	
H4A	−0.108849	0.130433	0.317815	0.032*	
H4B	−0.037111	0.325477	0.333300	0.032*	
C4A	0.1392 (3)	0.2023 (3)	0.28289 (7)	0.0214 (5)	
C5	0.0715 (3)	0.2181 (4)	0.23438 (8)	0.0304 (6)	
H5	−0.060695	0.228797	0.228874	0.036*	
C6	0.1933 (4)	0.2184 (4)	0.19427 (8)	0.0315 (6)	
H6	0.144780	0.230122	0.161566	0.038*	
C7	0.3856 (4)	0.2017 (4)	0.20182 (8)	0.0309 (6)	
H7	0.469721	0.200425	0.174352	0.037*	
C8	0.4550 (3)	0.1869 (3)	0.24957 (8)	0.0282 (5)	
H8	0.587475	0.176093	0.254702	0.034*	
C8A	0.3337 (3)	0.1876 (3)	0.29031 (7)	0.0194 (5)	
C9	0.6220 (3)	0.8178 (3)	0.43005 (7)	0.0163 (4)	
C10	0.6944 (3)	0.6256 (3)	0.43360 (7)	0.0150 (4)	
H10	0.659059	0.561013	0.402479	0.018*	
C11	0.6005 (3)	0.5311 (3)	0.47767 (7)	0.0149 (4)	
H11	0.460966	0.528552	0.471655	0.018*	
C12	0.6706 (3)	0.3389 (3)	0.47987 (7)	0.0151 (4)	
N2	0.2809 (3)	0.2123 (3)	0.38108 (7)	0.0226 (4)	
H2A	0.263 (4)	0.333 (3)	0.3823 (9)	0.029 (7)*	
H2B	0.326 (3)	0.179 (3)	0.4103 (7)	0.024 (6)*	
O1	0.7420 (2)	0.93738 (19)	0.43712 (6)	0.0257 (4)	
O2	0.4493 (2)	0.8404 (2)	0.42043 (5)	0.0183 (3)	
O3	0.8937 (2)	0.6193 (2)	0.43902 (6)	0.0204 (3)	
H3	0.933 (4)	0.721 (3)	0.4501 (9)	0.039 (8)*	
O4	0.6332 (2)	0.6239 (2)	0.52224 (5)	0.0206 (3)	
H4	0.743 (3)	0.609 (4)	0.5316 (10)	0.041 (8)*	
O6	0.7472 (2)	0.2755 (2)	0.51597 (5)	0.0242 (4)	
O5	0.6396 (2)	0.25433 (19)	0.43803 (5)	0.0181 (3)	
H5A	0.675 (4)	0.141 (3)	0.4404 (11)	0.055 (9)*	
O7	0.2156 (3)	0.5762 (2)	0.38150 (6)	0.0290 (4)	
H7A	0.296 (4)	0.651 (4)	0.3947 (11)	0.050 (9)*	
H7B	0.108 (4)	0.581 (5)	0.3967 (11)	0.063 (11)*	

### 1,2,3,4-Tetrahydroisoquinolin-2-ium (2S,3S)-3-carboxy-2,3-dihydroxypropanoate monohydrate Atomic displacement parameters (Å^2^)

**Table:**

	*U*^11^	*U*^22^	*U*^33^	*U*^12^	*U*^13^	*U*^23^
C1	0.0193 (11)	0.0331 (13)	0.0192 (10)	0.0045 (11)	−0.0019 (9)	0.0004 (9)
C3	0.0244 (13)	0.0404 (15)	0.0208 (10)	−0.0100 (12)	0.0004 (10)	0.0047 (10)
C4	0.0197 (12)	0.0395 (15)	0.0218 (11)	−0.0036 (11)	0.0000 (9)	0.0009 (10)
C4A	0.0236 (12)	0.0214 (11)	0.0190 (10)	−0.0013 (10)	−0.0003 (9)	0.0004 (8)
C5	0.0227 (12)	0.0440 (15)	0.0244 (11)	0.0006 (12)	−0.0051 (9)	0.0025 (10)
C6	0.0367 (14)	0.0433 (16)	0.0145 (10)	0.0000 (13)	−0.0045 (10)	0.0014 (10)
C7	0.0317 (13)	0.0413 (15)	0.0197 (11)	−0.0019 (13)	0.0062 (10)	−0.0009 (10)
C8	0.0221 (12)	0.0389 (15)	0.0235 (11)	0.0007 (11)	0.0011 (9)	−0.0017 (10)
C8A	0.0225 (11)	0.0188 (11)	0.0168 (9)	0.0003 (9)	−0.0021 (8)	−0.0012 (8)
C9	0.0179 (10)	0.0152 (10)	0.0159 (9)	0.0004 (9)	0.0013 (8)	−0.0004 (8)
C10	0.0124 (10)	0.0144 (10)	0.0182 (9)	0.0000 (8)	−0.0001 (8)	−0.0016 (8)
C11	0.0137 (10)	0.0169 (10)	0.0141 (9)	−0.0011 (8)	−0.0019 (8)	−0.0024 (8)
C12	0.0130 (10)	0.0175 (10)	0.0148 (9)	−0.0022 (8)	0.0011 (8)	0.0013 (8)
N2	0.0232 (10)	0.0298 (12)	0.0149 (8)	−0.0029 (9)	−0.0012 (8)	0.0027 (8)
O1	0.0202 (8)	0.0126 (7)	0.0443 (9)	0.0000 (7)	−0.0035 (8)	−0.0019 (7)
O2	0.0149 (7)	0.0195 (8)	0.0204 (7)	0.0019 (6)	0.0000 (6)	0.0001 (6)
O3	0.0141 (7)	0.0140 (8)	0.0331 (8)	0.0007 (6)	0.0014 (7)	−0.0021 (6)
O4	0.0169 (8)	0.0255 (8)	0.0194 (7)	0.0031 (7)	−0.0023 (7)	−0.0090 (6)
O6	0.0279 (8)	0.0260 (9)	0.0187 (7)	0.0051 (8)	−0.0050 (6)	0.0031 (6)
O5	0.0240 (8)	0.0116 (8)	0.0188 (7)	0.0018 (6)	−0.0029 (6)	−0.0008 (6)
O7	0.0222 (9)	0.0336 (10)	0.0312 (9)	−0.0026 (8)	0.0034 (8)	−0.0108 (8)

### 1,2,3,4-Tetrahydroisoquinolin-2-ium (2S,3S)-3-carboxy-2,3-dihydroxypropanoate monohydrate Geometric parameters (Å, º)

**Table:**

C1—N2	1.483 (3)	C8—H8	0.9500
C1—C8A	1.510 (3)	C9—O1	1.247 (2)
C1—H1A	0.9900	C9—O2	1.260 (3)
C1—H1B	0.9900	C9—C10	1.529 (3)
C3—N2	1.489 (3)	C10—O3	1.417 (2)
C3—C4	1.518 (3)	C10—C11	1.534 (3)
C3—H3A	0.9900	C10—H10	1.0000
C3—H3B	0.9900	C11—O4	1.407 (2)
C4—C4A	1.512 (3)	C11—C12	1.522 (3)
C4—H4A	0.9900	C11—H11	1.0000
C4—H4B	0.9900	C12—O6	1.211 (2)
C4A—C8A	1.393 (3)	C12—O5	1.312 (2)
C4A—C5	1.397 (3)	N2—H2A	0.91 (2)
C5—C6	1.382 (3)	N2—H2B	0.883 (19)
C5—H5	0.9500	O3—H3	0.87 (2)
C6—C7	1.380 (3)	O4—H4	0.82 (2)
C6—H6	0.9500	O5—H5A	0.89 (2)
C7—C8	1.382 (3)	O7—H7A	0.87 (2)
C7—H7	0.9500	O7—H7B	0.87 (2)
C8—C8A	1.393 (3)		
			
N2—C1—C8A	112.07 (18)	C8A—C8—H8	119.5
N2—C1—H1A	109.2	C4A—C8A—C8	119.6 (2)
C8A—C1—H1A	109.2	C4A—C8A—C1	122.13 (19)
N2—C1—H1B	109.2	C8—C8A—C1	118.22 (19)
C8A—C1—H1B	109.2	O1—C9—O2	126.43 (19)
H1A—C1—H1B	107.9	O1—C9—C10	115.97 (17)
N2—C3—C4	108.99 (18)	O2—C9—C10	117.60 (17)
N2—C3—H3A	109.9	O3—C10—C9	111.74 (16)
C4—C3—H3A	109.9	O3—C10—C11	109.57 (16)
N2—C3—H3B	109.9	C9—C10—C11	109.73 (16)
C4—C3—H3B	109.9	O3—C10—H10	108.6
H3A—C3—H3B	108.3	C9—C10—H10	108.6
C4A—C4—C3	112.13 (19)	C11—C10—H10	108.6
C4A—C4—H4A	109.2	O4—C11—C12	112.33 (16)
C3—C4—H4A	109.2	O4—C11—C10	111.24 (16)
C4A—C4—H4B	109.2	C12—C11—C10	108.98 (16)
C3—C4—H4B	109.2	O4—C11—H11	108.1
H4A—C4—H4B	107.9	C12—C11—H11	108.1
C8A—C4A—C5	118.6 (2)	C10—C11—H11	108.1
C8A—C4A—C4	120.63 (19)	O6—C12—O5	125.21 (19)
C5—C4A—C4	120.8 (2)	O6—C12—C11	123.19 (18)
C6—C5—C4A	121.3 (2)	O5—C12—C11	111.59 (16)
C6—C5—H5	119.4	C1—N2—C3	112.25 (17)
C4A—C5—H5	119.4	C1—N2—H2A	109.3 (17)
C7—C6—C5	119.9 (2)	C3—N2—H2A	108.7 (18)
C7—C6—H6	120.1	C1—N2—H2B	110.2 (16)
C5—C6—H6	120.1	C3—N2—H2B	109.1 (16)
C6—C7—C8	119.6 (2)	H2A—N2—H2B	107 (2)
C6—C7—H7	120.2	C10—O3—H3	109.0 (19)
C8—C7—H7	120.2	C11—O4—H4	110 (2)
C7—C8—C8A	121.0 (2)	C12—O5—H5A	111 (2)
C7—C8—H8	119.5	H7A—O7—H7B	110 (3)
			
N2—C3—C4—C4A	49.8 (3)	N2—C1—C8A—C8	167.3 (2)
C3—C4—C4A—C8A	−18.9 (3)	O1—C9—C10—O3	−6.6 (2)
C3—C4—C4A—C5	161.3 (2)	O2—C9—C10—O3	173.40 (17)
C8A—C4A—C5—C6	0.3 (4)	O1—C9—C10—C11	115.09 (19)
C4—C4A—C5—C6	−179.8 (2)	O2—C9—C10—C11	−64.9 (2)
C4A—C5—C6—C7	0.4 (4)	O3—C10—C11—O4	66.1 (2)
C5—C6—C7—C8	−0.8 (4)	C9—C10—C11—O4	−57.0 (2)
C6—C7—C8—C8A	0.3 (4)	O3—C10—C11—C12	−58.3 (2)
C5—C4A—C8A—C8	−0.8 (4)	C9—C10—C11—C12	178.67 (15)
C4—C4A—C8A—C8	179.4 (2)	O4—C11—C12—O6	−0.7 (3)
C5—C4A—C8A—C1	−179.8 (2)	C10—C11—C12—O6	123.1 (2)
C4—C4A—C8A—C1	0.4 (4)	O4—C11—C12—O5	−179.82 (16)
C7—C8—C8A—C4A	0.4 (4)	C10—C11—C12—O5	−56.1 (2)
C7—C8—C8A—C1	179.5 (2)	C8A—C1—N2—C3	46.4 (3)
N2—C1—C8A—C4A	−13.7 (3)	C4—C3—N2—C1	−65.8 (3)

### 1,2,3,4-Tetrahydroisoquinolin-2-ium (2S,3S)-3-carboxy-2,3-dihydroxypropanoate monohydrate Hydrogen-bond geometry (Å, º)

**Table:**

*D*—H···*A*	*D*—H	H···*A*	*D*···*A*	*D*—H···*A*
C1—H1*A*···O2^i^	0.99	2.53	3.263 (3)	131
C3—H3*A*···O1^ii^	0.99	2.63	3.343 (3)	129
N2—H2*A*···O7	0.91 (2)	1.85 (2)	2.762 (3)	176 (2)
N2—H2*B*···O6^iii^	0.88 (2)	2.09 (2)	2.787 (2)	135 (2)
N2—H2*B*···O5	0.88 (2)	2.41 (2)	2.981 (2)	123 (2)
O3—H3···O1	0.87 (2)	2.14 (3)	2.612 (2)	114 (2)
O3—H3···O4^iv^	0.87 (2)	1.98 (2)	2.766 (2)	151 (3)
O4—H4···O2^iv^	0.82 (2)	1.99 (2)	2.730 (2)	150 (3)
O5—H5*A*···O1^i^	0.89 (2)	1.60 (2)	2.480 (2)	173 (3)
O7—H7*A*···O2	0.87 (2)	1.91 (2)	2.782 (2)	173 (3)
O7—H7*B*···O3^v^	0.87 (2)	1.92 (2)	2.772 (2)	169 (3)

Symmetry codes: (i) *x*, *y*−1, *z*; (ii) *x*−1, *y*−1, *z*; (iii) *x*−1/2, −*y*+1/2, −*z*+1; (iv) *x*+1/2, −*y*+3/2, −*z*+1; (v) *x*−1, *y*, *z*.
